# Clinical evaluation of a biomechanical guidance system for periacetabular osteotomy

**DOI:** 10.1186/s13018-016-0372-3

**Published:** 2016-03-30

**Authors:** Ryan J. Murphy, Robert S. Armiger, Jyri Lepistö, Mehran Armand

**Affiliations:** Research and Exploratory Development Department, Johns Hopkins University Applied Physics Laboratory, 11100 Johns Hopkins Rd, Laurel, MD 20723 USA; Department of Mechanical Engineering, Johns Hopkins University, Baltimore, MD USA; Orton Orthopaedic Hospital, Helsinki, Finland

**Keywords:** Periacetabular osteotomy, Computer-assisted surgery, Dysplasia, Biomechanics

## Abstract

**Background:**

Populations suffering from developmental dysplasia of the hip typically have reduced femoral coverage and experience joint pain while walking. Periacetabular osteotomy (PAO) is one surgical solution that realigns the acetabular fragment. This challenging surgery has a steep learning curve. Existing navigation systems for computer-assisted PAO neither track the released fragment nor offer the means to assess fragment location. An intraoperative workstation—the biomechanical guidance system (BGS)—developed for PAO incorporates intraoperative fragment tracking and acetabular characterization through radiographic angles and joint biomechanics. In this paper, we investigate the accuracy and effectiveness of the BGS for bone fragment tracking and acetabular characterization in clinical settings as compared to conventional techniques and postoperative assessments. We also report the issues encountered and our remedies when using the BGS in the clinical setting.

**Methods:**

Eleven consecutive patients (aged 22–48, mean 34, years) underwent 12 PAO surgeries (one bilateral surgery) where the BGS collected information on acetabular positioning. These measurements were compared with postoperative CT data and manual measurements made intraoperatively.

**Results:**

No complications were reported during surgery, with surgical time—95–210 (mean 175) minutes—comparable to reported data for the conventional approach. The BGS-measured acetabular positioning showed strong agreement with postoperative CT measurements (−0.3–9.2, mean 3.7, degrees), whereas larger differences occurred between the surgeon’s intraoperative manual measurements and postoperative CT measurements (−2.8–21.3, mean 10.5, degrees).

**Conclusions:**

The BGS successfully tracked the acetabular fragment in a clinical environment without introducing complications to the surgical workflow. Accurate 3D positioning of the acetabulum may provide more information intraoperatively (e.g., anatomical angles and biomechanics) without adversely impacting the surgery to better understand potential patient outcomes.

## Background

Periacetabular osteotomy (PAO) is a hip preservation surgery performed to treat congenital or development deformity of the acetabulum, such as that observed in developmental dysplasia of the hip (DDH). The PAO surgery aims at improving poor femoral coverage by reorienting the acetabulum and stabilizing the hip joint [1]. After patient examination and preoperative imaging (e.g., standing anteroposterior and lateral X-ray images, and computed tomography scans), surgeons plan adjustments to the acetabular fragment to achieve coverage observed in normal hips [2–6]. The PAO, introduced by Ganz in 1988 [1], has become one common procedure addressing adult hip dysplasia. Unfortunately, this procedure has a steep learning curve [7–10]. Surgeons not only have to successfully release the fragment (while maintaining integrity of the joint and pelvic ring), they must effectively realign the fragment without introducing further complications. Overcorrection can lead to femoroacetabular impingement and reduced range of motion [8–15]; under correction may not effectively reduce the pain or discomfort of the patient. In either case, there is the potential to perform revision operations, e.g., a total hip arthroplasty [16]. As such, surgeons with more information (e.g., experience, intraoperative feedback) will likely be able to better correct the fragment and reduce complications.

Intraoperative feedback (conventionally fluoroscopy) comparing the current surgical state to the plan is limited during surgery. When addressing fragment realignment, surgeons gauge several radiographic parameters, including the center-edge (CE) angle of Wiberg [6], the acetabular index (AC) angle of Tonnis [17], and continuity of the Shenton line. Typical corrections result in a CE of 25°–30° and AC angles of 0°. C-arm positioning and patient alignment can affect these radiographic measurements. For 3D acetabular realignment, surgeons rely on visual estimates from limited intraoperative 2D imaging (i.e., X-ray images), surgical experience, and limited means of measuring and/or verifying the planned alignment of the osteotomized fragment in all three dimensions using external hardware (e.g., Kirschner wires (K-wires)) [18].

Several studies have proposed computer-assisted surgery for PAO (e.g., [19–24]) and described a number of potential benefits, including preoperative planning, and visual feedback combined with intraoperative navigation. However, the main limitation of each system is either the lack of fragment tracking [19–22, 24] or the inability to intraoperatively assess fragment location [21, 22]. Moreover, several inherent surgical challenges limit exact execution of the preoperative plan for PAO. Among these challenges are the variations of the osteotomy line due to bone movement resulting from hammering an osteotome, and the variable constraint forces imposed by soft tissues during fragment realignment. Therefore, the ability to intraoperatively analyze the state of surgery and update the preoperative plan is especially important for PAO. This set of attributes is currently not offered by other systems.

To address these limitations, we developed the biomechanical guidance system (BGS) [25–30]. The BGS combines preoperative planning with intraoperative fragment tracking, plan updates, and acetabular characterization through radiographic angles and near real-time biomechanics. This study assessed the impact of using the BGS on the surgical approach (e.g., usability and length of surgery), and considered its effectiveness in identifying the intraoperative position of the acetabular fragment compared to both conventional techniques and postoperative evaluation.

## Methods

In this study, the operating surgeon (JL) consecutively performed 12 PAOs on 11 patients (including a bilateral PAO) using the protocol established at Orton Orthopaedic Hospital in Helsinki, Finland, and Johns Hopkins University (approved by JHM IRB #NA_00001257) between November 2005 and November 2009. The patient cohort consisted of three males (one of whom had PAO performed for each hip in separate operations) and eight females, with a total of six operations on the left hips and six on right. The patients were 22–48 (mean 34) years of age, weighing 25–87 (mean 59) kilograms. Per BGS protocol, we conducted pelvic CT scans of each patient prior to surgery. The preoperative CT scans were conducted on a PQ2000 (Picker International, Inc.) with slice thickness less than 4 mm and spacing between slices of less than 2 mm. Patients with concurrent femoral pathologies such as slipped capital femoral epiphysis or Legg-Calve-Perthes syndrome were excluded from the study. All patients reported complaints about frequent hip pain as an indication for surgery.

The preoperative data preparation protocol followed that described in [29]. Briefly, the preoperative CT scans were resampled to 1 mm slice thickness, segmented using image processing software (Amira, Visage Imaging; Berlin, Germany), and placed into a common coordinate frame consistent with that described by Bergmann et al. [31]. The acetabular rim was segmented from the CT to generate a contact surface [26]. During this procedure, points along the acetabular rim were digitized on oblique CT reformats rotated at 7.5° increments about the medio-lateral axis of the pelvis. Per standard surgical protocol at the Orton Hospital, the surgeon and radiologists analyzed standing AP radiographs and CT slices to assess the degree of dysplasia. From the images, the surgeon developed a plan for the reorientation. After planning, the surgeon performed a Ganz osteotomy on the patient [29].

**Fig. 1:**
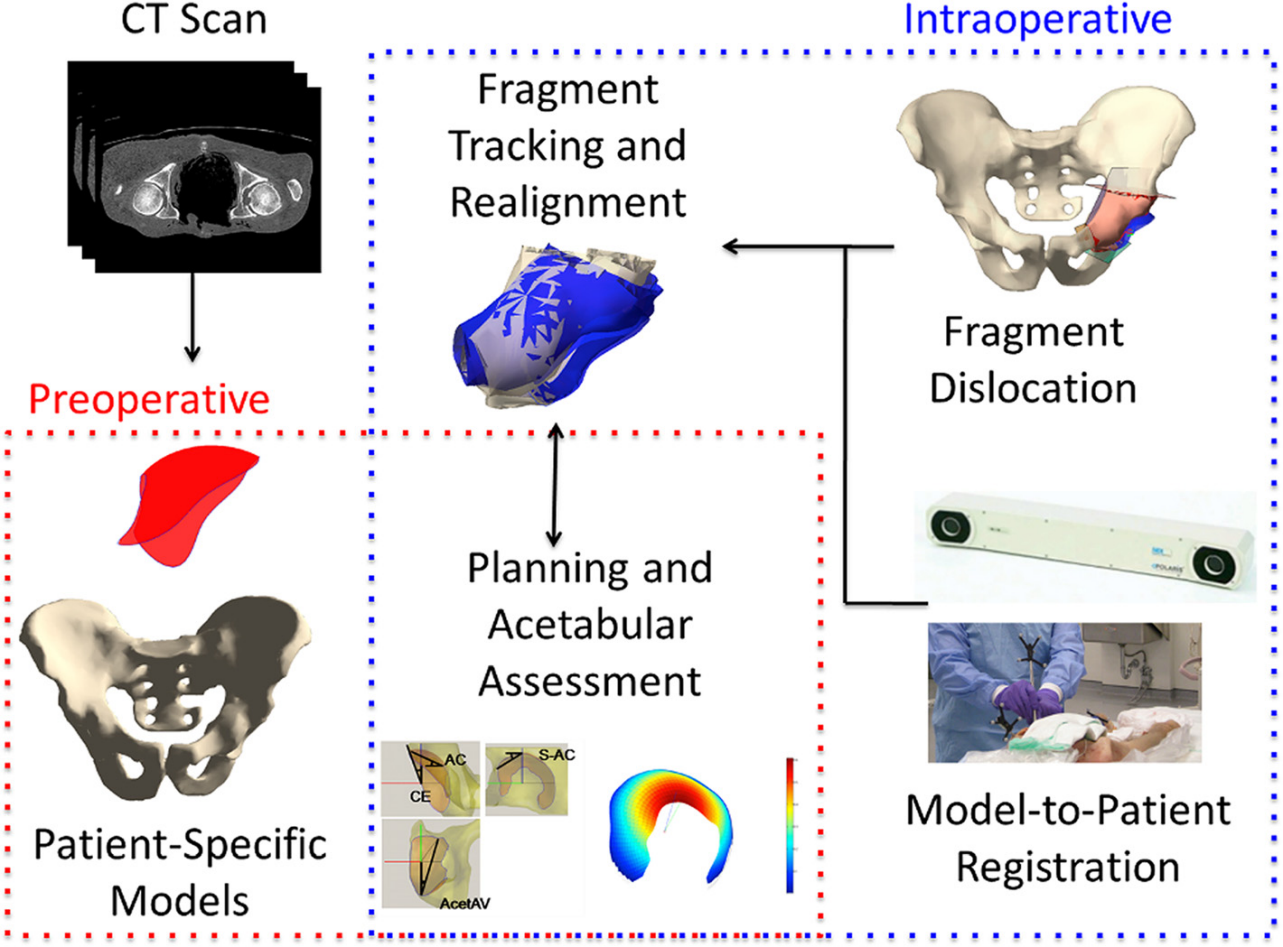
BGS procedure overview. The BGS analyzes patient-specific models of the pelvis and acetabulum from preoperative CT data to report on the acetabular characterization (contact pressure and radiographic angles) from which the surgeon details the planned realignment. Intraoperatively, the BGS tracks the fragment location, providing feedback to the surgeon regarding the current acetabular characterization and proximity to the planned realignment

**Fig. 2:**
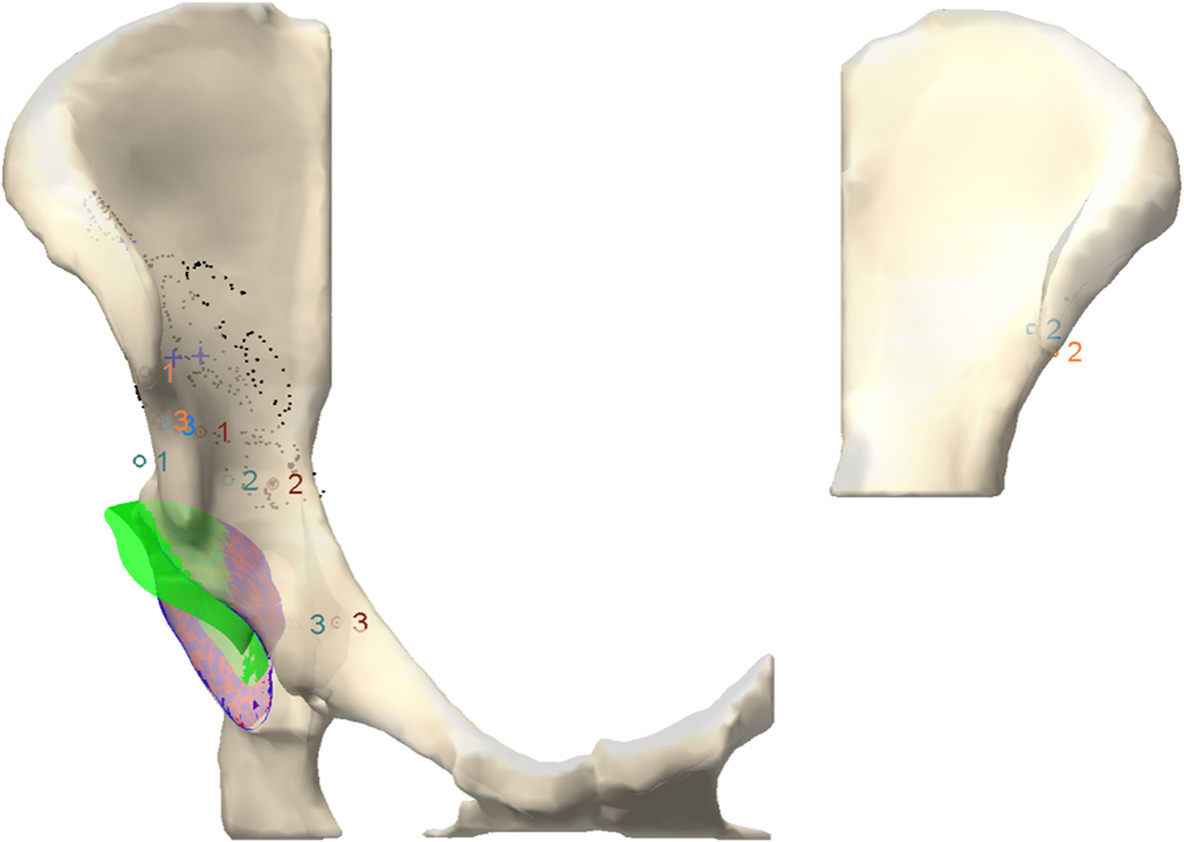
Example of the BGS intraoperative data collection during PAO. CT landmarks (*red 1*, *2*, *3*) and the associated digitized patient landmarks (*blue 1*, *2*, *3*) provide a gross registration. Surface points (*black dots*) refine the registration while confidence points (*blue* ‘*+*’ symbol) provide a virtual reference. The fragment points digitized prior to (*red 1*, *2*, *3*) and after mobilization (*teal 1*, *2*, *3*) track the realigned acetabulum (*green*) compared to the initial position (*blue*/*red fragment*)

During the osteotomy, per IRB protocol, the surgeon performed the surgeries using his conventional method while using the BGS to only collect intraoperative measurements for postoperative comparisons. The BGS uses an infrared tracker (Polaris, NDI, Inc., Waterloo, CA) to digitize points and record data during surgery (Fig. 1). Since we integrated BGS data collection with the surgery, the procedure is slightly modified, as explained in [29]. Following a stab incision on the iliac crest, the surgeon attached a removable rigid body (RB) to the contralateral iliac crest using an anchoring pin. Prior to any osteotomy, the surgeon digitized three landmarks on the pelvis (the ASIS and AIIS on the ipsilateral side, and the ASIS on the contralateral side) that were previously defined in the CT model. After osteotomizing the anterior inferior iliac spine as part of the exposure, a bone burr created a set of four 1.5 mm divots on the iliac cortex (Fig. 2). These references served as confidence points for the pelvis throughout the surgery. Under normal circumstances, these points are at a constant location relative to the navigation system. If, however, the navigation markers shift unintentionally, the confidence points help to reestablish the system calibration. Next, the surgeon digitized a set of points more broadly by moving the digitizer across the exposed surfaces of the iliac face and crest. Either an iterative closest point (ICP) [32] or an unscented Kalman filter (UKF) [33] registered the collected points to the patient’s anatomy. Once the registration frame was established, the BGS displayed the motion of the navigation tools with respect to the computer-rendered pelvis in real time.

After the initial data collection and registration, the surgeon made two osteotomy cuts (upper and medial). Next, four additional bone burrs were created on the fragment (Fig. 2). Before digitizing these points, the surgeon first digitized the confidence points. In the surgeries performed under this study, the surgeon also used his conventional tracking method by attaching K-wires to the fragment [18]. The surgeon fully released the acetabular fragment and partially fixed it into position. At each partial fixation, the surgeon measured the K-wire positions using a goniometer, the confidence points were digitized to update the patient frame, and the fragment points were digitized to update the acetabulum’s position. From the tracker information, the BGS defined the fragment’s transformation and analyzed the position (Fig. 2). Once the acetabular fragment was successfully positioned, the fragment was fully fixed to the bone using bone screws and a final measurement of the acetabular position was recorded to represent the total hip joint realignment achieved from the PAO.

Postoperative CT scans were conducted at least 4 months after surgery and used as the ground truth for comparison of planned and intraoperative measurements of the fragment realignment. Patients were interviewed and asked to answer a questionnaire to obtain the Q scores [34]. Each patient was also interviewed in August 2011 to understand the current condition of the hip and determine if any subsequent surgeries were performed.

Radiographic angles are commonly used to assess the results of PAO. Previous work compared automated measurement of CT angles to observer-measured angles and found minimal discrepancies within a single modality [26]. However, angles measured from CT slices have limitations representing the anatomy from only a thin cross section and are sensitive to the particular image slice selected. As such, we performed simulated radiographic measurements [29] in addition to the CT angle computation [26]. The simulated radiographic technique projects the segmented acetabulum onto a virtual image to create a C-arm view. From this view, the algorithm automatically defines the most lateral point on the acetabular rim and the most medial aspect of the sourcil to compute the center-edge (CE) angle using the definition of Wiberg [6] and the acetabular index (AC) angle defined by Tonnis [17].

To validate the BGS tracking, we compared intraoperative measurements to postoperative CT scans taken at least 4 months after each surgery. First, we aligned the preoperative and postoperative pelvis CT scans, with the operative region masked out, using a normalized mutual information (NMI) registration technique implemented in commercial software (Amira, FEI Visualization Sciences Group, Burlington, MA). The realigned acetabular cup was segmented from the postoperative CT and compared with the preoperative segmentation through a registration using the UKF algorithm [33]. The resulting transformation was used as the ground truth for the intraoperative measurement.

**Fig. 3:**
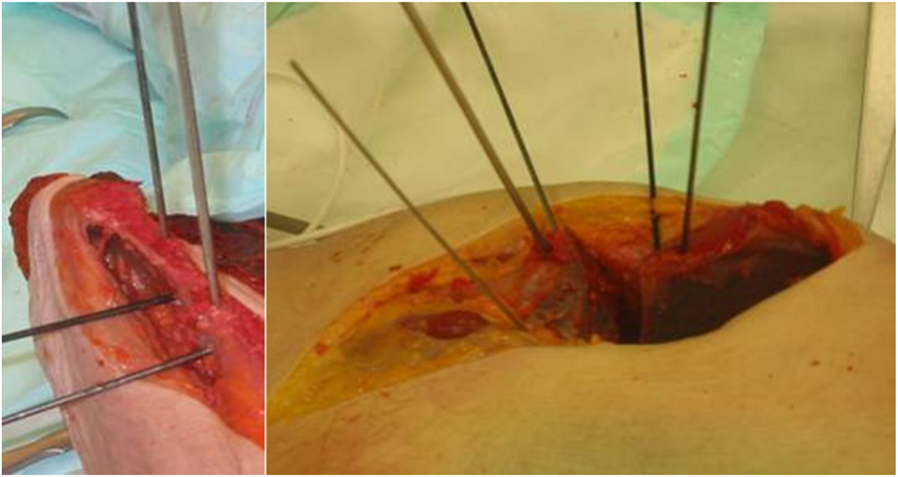
Surgical K-wires used to measure the fragment realignment

We also compared the surgeon’s intraoperative K-wire measurements to the intraoperative BGS measurements and the postoperative measurements. The K-wires helped to measure the acetabular rotation angles with respect to the operating room. To simulate these same angles for direct comparison, the intraoperative and postoperative transformations measured from the BGS were projected to the anatomical planes (ab-adduction in the frontal plane, flexion-extension in the sagittal plane, and ante-retroversion in the transverse plane) to allow an appropriate comparison between the techniques (Fig. 3).

The Kruskal–Wallis one-way analysis of variance (ANOVA) compared the projected measurement technique (K-wires, intraoperative, and postoperative) for each type of measurement. If necessary, the Tukey honest significant difference test identified which measurement techniques were significantly different. A Wilcoxon rank sum test identified any differences in radiographic projection measurements computed intraoperatively and postoperatively. Any *p* values less than 0.05 were considered significant. Statistical computations were performed using Matlab.

## Results

Overall surgical time was 95–210 (mean 175) minutes. The BGS data acquisition did not introduce any substantial difficulties for the surgeon. As we encountered minor difficulties in surgery with the BGS, we increased the robustness of the system. In particular, during the surgery for patient 2, the reference markers shifted (rotated about the axis of the mounting screw). Since the axis of rotation was known, we accounted for this rotation angle in postoperative analyses. During the surgery for patient 5, the osteotomy cut split one of the fragment bone burr points after recording the first realignment; in this case, the last known position of the acetabulum was used for analysis.

Follow-up evaluations on the patients showed that 2 of 11 patients underwent subsequent revision procedures for complications unrelated to BGS data acquisition. Patient 8 underwent THA for pain 1 year after surgery, and patient 10 had the fixation screws removed and an uneven edge of the anterior acetabulum corrected 10 months after surgery. The lowest 5-year Q score (Table 1) reported was 69 (patients 6 and 10).

**Table 1:** Patient postoperative Q scores. No Q score was available for patient 8 (revision to THA)

Patient	1	2	3	4	5	6	7	8	9	10	11	12
Q score	100	91	85	92	92	69	96	–*	97	69*	99	97

Patients identified with an asterisk (8, 10) underwent subsequent surgery within 1 year of the PAO operation. All Q scores were obtained in August 2011

The positional measurements indicated substantial differences between the surgeon’s perceived measurements (via K-wire measurements) and the intraoperative/postoperative projected measurements (Tables 2 and 3). The Kruskal–Wallis ANOVA identified significant differences in measurement techniques for the adduction angle (*p* = 0.014). The average difference between the K-wire and the postoperative measured adduction angle was −2.8–21.3 (mean 10.5) degrees compared to the difference between the intraoperative and postoperative adduction angle of −0.3–9.2 (mean 3.7) degrees (Table 3). The anteversion angle exhibited a significant difference between K-wire and either intraoperative or postoperative measurements (*p* < 0.001), with the intraoperative and postoperative measurements exhibiting a −6.2–4.4 (mean −0.5) degree difference. However, there was no significant difference in measuring the extension angle (*p* = 0.47) even with a −8.1–11.2 (mean −0.1) degree difference between intraoperative and postoperative measures and a −7.6–19.9 (mean 4.2) degree difference between K-wires and postoperative measurements.

**Table 2:** Projective angle measurements (in degrees) made using K-wires (intraoperatively), the BGS (intraoperatively), and postoperative CT reformats

	1	2	3	4	5	6	7	8	9	10	11	12
K-wires												
Adduction	15	8	40	25	25	20	20	35	15	20	12	20
Extension	10	0	25	10	7	23	12	20	0	5	10	15
Anteversion	3	0	5	0	0	0	−20	0	0	3	0	0
Intraoperative												
Adduction	13	17	28	14	12	17	5	18	14	17	9	20
Extension	6	2	1	4	7	10	15	17	2	5	2	8
Anteversion	9	6	8	5	1	4	8	10	6	3	1	11
Postoperative												
Adduction	12	11	19	13	10	8	2	19	8	17		
Extension	3	3	5	3	15	12	3	15	3	8		
Anteversion	6	6	11	1	7	6	5	13	7	3		

Note that patients 11 and 12 did not undergo postoperative CT

**Table 3:** Average differences between projective angle measurements made using K-wires (intraoperatively), the BGS (intraoperatively), and postoperative CT reformats (in degrees)

	K-wires—intraoperative	Intraoperative–postoperative	K-wires—postoperative
Adduction	6.7 (−8.6–16.5)	3.7 (−0.3–9.2)	10.5 (−2.8–21.3)
Extension	4.3 (−2.7–24.5)	−0.1 (−8.1–11.2)	4.2 (−7.6–19.9)
Anteversion	−7.0 (−27.7 to −0.4)	−0.5 (−6.2–4.4)	−7.5 (−24.5–0.01)

**Fig. 4:**
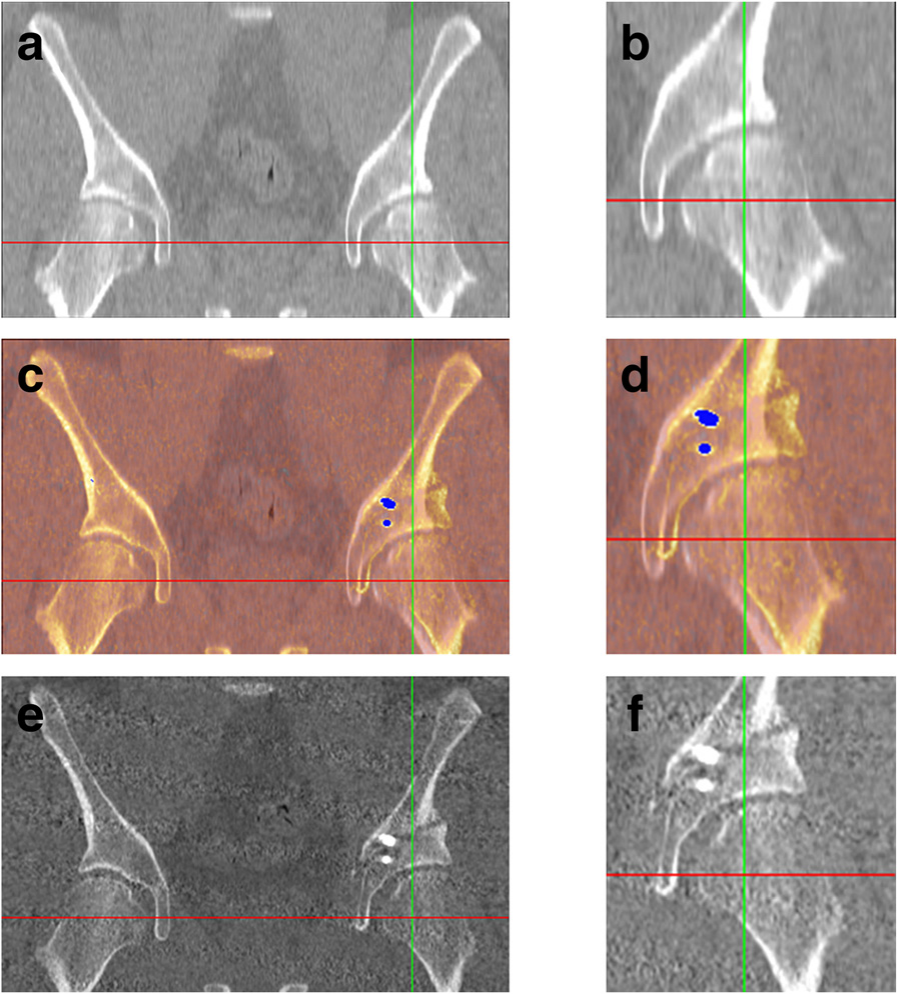
Example of the pre- and postoperative axial CT slices. The CE angle measured automatically from X-ray projections changed from 21° preoperatively to 29° postoperatively. **a** Preoperative CT slice and **b** a zoomed in region about the acetabulum. **c** Preoperative CT slice with a postoperative overlay and **d** a zoomed in region about the acetabulum. In these displays, the postoperative was highlighted with a color-based filter to distinguish it from preoperative; the *blue dots* are coloring effects due to the intensity of the fixation screws. **e** Postoperative CT slice and **f** a zoomed in region about the acetabulum

The radiographic projection angles indicated strong agreement between the intraoperative and postoperative measurements (Table 4). The average change of radiographic angles from preoperative to postoperative was 0.4–28.4 (mean 14.8) for CE and −21.8–2 (mean −12.6) for AC (Fig. 4). The Wilcoxon rank sum test identified no significant differences between angles measured intraoperatively with the BGS or postoperatively (*p* = 0.68 for CE and *p* = 0.57 for AC). The difference between postoperative and intraoperative measurements was −6.1–3.6 (mean −1.8) degrees for CE and −3.5–7.7 (mean 2.5) degrees for AC.

**Table 4:** Radiographic angle automatically registered from the preoperative, intraoperative, and postoperative data

		1	2	3	4	5	6	7	8	9	10	11	12
Preop	CE	25	29	−7	21	23	26	30	7	21	14	27	27
	AC	18	7	34	23	14	14	10	23	18	19	11	3
Intraop	CE	39	46	25	36	32	47	30	33	35	31	36	43
	AC	4	−11	6	8	6	−4	9	3	3	4	3	3
Postop	CE	38	40	19	34	36	44	30	35	29	30	–	–
	AC	5	−5	14	11	2	1	12	1	9	4	–	–

Note that patients 11 and 12 did not undergo postoperative CT

## Discussion

Periacetabular osteotomy is a challenging and demanding procedure. At present, no surgical tools exist that successfully couple a preoperative plan with intraoperative navigation and execution of that plan. This study investigated the BGS workstation’s ability to accurately and effectively track the acetabular bone fragment created during PAO. Overall, accuracy was found to be better than manual surgical measurements (K-wires), with strong agreement between postoperative CT measures and the intraoperative BGS measures. The system added no complications to surgery, with a surgical time comparable to conventional techniques. Accurate three-dimensional fragment tracking during PAO may add substantial benefit to surgeons, providing information not available during conventional surgery such as three-dimensional visualization, radiographic characterization from any view, and biomechanical analyses.

This work is limited by the small patient sample with, at most, 5-year follow-up. Future studies with a greater number of patients are necessary. The resampling of CT data may introduce errors into the virtual models. However, higher-resolution CT scans will dramatically increase radiation to the sensitive pelvic and reproductive areas of the patients, which is undesirable for the patient. An alternative approach could consider statistical atlas extrapolation of lower-resolution CT scans to reduce radiation exposure [35]. Additionally, it is difficult to define a ground truth from the postoperative data and compare with the intraoperative data. Errors are often not measurable and can occur at different steps. In particular, the sources of error include: (1) CT-to-CT (volumetric) registration to align the pre- and postoperative scans. (2) Mesh-to-mesh registration aligning the segmented acetabular lunate. (3) During the bone union phase, fragment fixation may change in the months after surgery before postoperative measurement. Therefore, these errors comparing the intraoperative and postoperative assessments are reasonable and expected.

We previously compared the computerized measurements with inter-observer variability among three observers when performing manual measurements of the above anatomical angles using both pre- and postoperative CT scans [26]. In that study, we reported that the mean difference between the computerized measurements and the control group (defined as the average manual measurements of three observers) was 1.3° over all measured angles. This was comparable to the mean and standard deviation of each of the observers when compared to the control group (average of the observers). For this reason, we used computerized measurements of the postoperative CT angles as ground truth.

The BGS did not introduce any adverse effects on the surgical routine in PAO. The intraoperative use of the BGS did not dramatically increase surgical time from established values for PAO [9, 14, 15, 36, 37]. There were two concerns in the BGS architecture throughout the testing that have been accounted for the subsequent revisions. While performing the procedure on patient 2, there was a rigid body shift for the pelvis rigid body. However, we estimated the transformation of the pelvis rigid body using an observed rotation, enabling recovery of all the surgical data. To mitigate future problems, we introduced the concept of confidence points taken during the CT-to-patient registration stage before osteotomy. Recording the confidence points both prior to and after osteotomy defines the change in the rigid body position updates the model-to-patient registration. Moreover, this technique allows the surgeon to remove the pelvis rigid body if it interferes with the surgery. The second problem during surgery occurred with patient 5. Here, one of the fragment landmarks was accidentally removed while making the osteotomies, resulting in the inability to track the fragment after the first partial fixation. This explains part of the large variation between the intraoperative and postoperative positions for this patient. Subsequently, the BGS procedure was modified to use four fragment landmarks for redundancy, and to create the lateral osteotomy before creating the bone burr to ensure a clear delineation of the pelvis and acetabular fragment.

**Fig. 5:**
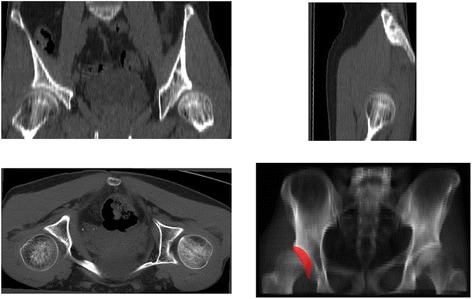
Example of severe dysplasia. Patient 3 exhibited severe dysplasia, as visible in the CT reformats and surface view. In the digitally-reconstructed AP radiograph, the acetabulum is highlighted

The differences between the registered postoperative and intraoperative CE and AC angles were well under 5° (−1.8° for CE and 2.5° for AC), indicating strong confidence in the measurements. Moreover, using the postoperative segmentation (i.e., a different acetabular rim trace than the intraoperative model), there were still only small errors between the postoperative and intraoperative measurements. Patients 2 and 3 exhibited the largest differences in radiographic angle computation (Table 3). It is likely that the differences observed in patient 2 are due to the loss of fully reliable tracking. Patient 3 was a highly dysplastic patient (Fig. 5), and small errors in tracking with this patient population (severe dysplasia with a very shallow cup) may lead to higher measurement errors. Using postoperative data as ground truth, there is better agreement with the intraoperative transformation than the surgeon’s manual measurements (Table 2). Assuming that the lateral change should roughly correlate with the change in CE and AC angles, manual measurements poorly predict the change in CE and AC angles. However, the BGS tracking showed strong agreement between the expected change and the measured change.

The results of BGS are in general agreement with other work reported on previous studies with computer-assisted PAO systems (e.g., [19–24]). Generally, these systems provide preoperative planning with visual assistance when performing the surgery. Studies on these systems have concluded that navigation and visualization aids offer several benefits, especially with regard to inexperienced surgeons. Moreover, the systems neither positively nor negatively affected the outcome of PAO. Hsieh et al. [20] showed that the radiographic correction, and functional outcome was comparable between navigated and conventional techniques with an experienced surgeon. Langlotz et al. [21, 22] experienced slightly higher surgical time occurring during the operation, but noted no significant impacts.

## Conclusions

These observations and data support to the need for the BGS. The BGS did not add a significant increase the length of surgery (on the order of 3 to 5 min for digitization procedures and 5 to 7 min total since expensive computations such as the registration are performed while the surgeon is operating). Moreover, fragment tracking enables a realistic, repeatable estimation of the fragment location and subsequent acetabular alignment. These positional estimates are more accurate than the manual method and provide additional information unavailable in fluoroscopic images (i.e., horizontal and sagittal planes). To conclude, the BGS can safely provide a surgeon three-dimensional geometric and biomechanical information before and during surgery regarding the predicted successes of the PAO.

### Ethics approval and consent to participate

This study was approved under JHMI IRB #05-09-02-01, and all patients gave consent.

## Abbreviations

AC: acetabular index
AIIS: anterior inferior iliac spine
ASIS: anterior superior iliac spine
BGS: biomechanical guidance system
CE: center edge
DDH: developmental dysplasia of the hip
ICP: iterative closest point
PAO: periacetabular osteotomy
RB: rigid body
UKF: unscented Kalman filter
